# Age-Dependent Hematologic Toxicity Profiles and Prognostic Serologic Markers in Postoperative Radiochemotherapy Treatment for Uterine Cervical Cancer

**DOI:** 10.1177/15330338221118188

**Published:** 2022-08-10

**Authors:** Eva Meixner, Line Hoeltgen, Philipp Hoegen, Laila König, Nathalie Arians, Laura L. Michel, Katharina Smetanay, Carlo Fremd, Andreas Schneeweiss, Jürgen Debus, Juliane Hörner-Rieber

**Affiliations:** 1 Department of Radiation Oncology, 27178 Heidelberg University Hospital, Heidelberg, Germany; 2 Heidelberg Institute of Radiation Oncology (HIRO), Heidelberg, Germany; 3 National Center for Tumor diseases (NCT), Heidelberg, Germany; 4 Department of Gynecology and Obstetrics, 9144 Heidelberg University Hospital, Heidelberg, Germany; 5 Heidelberg Ion Therapy Center (HIT), Heidelberg, Germany; 6 Clinical Cooperation Unit Radiation Oncology, German Cancer Research Center (DKFZ), Heidelberg, Germany

**Keywords:** hematotoxicity, anemia, leukopenia, thrombocytopenia, gynecological neoplasm, adjuvant treatment, chemoradiation, systemic inflammatory markers

## Abstract

**Introduction:** In the adjuvant setting for cervical cancer, classical risk factors for postoperative radiochemotherapy have been established. However, data on laboratory changes during therapy and the prognostic value of serological markers are limited and further knowledge is needed to optimize the toxic trimodal regimen. **Methods:** We retrospectively identified 69 women who underwent weekly postoperative radiochemotherapy with 40 mg/m^2^ of cisplatin for cervical cancer between 2010 and 2021 at a single center. Laboratory parameters were recorded before, at each cycle and after radiochemotherapy. Kaplan-Meier and log-rank analyses were used to calculate and compare survival, groups were compared using the Mann–Whitney *U*, χ^2^, and variance tests. **Results:** With a median follow-up of 17.7 months, the 1- and 5-year local control rates were 94.0% and 73.7%, respectively, with significantly better rates for more chemotherapy cycles and negative resection margins. Only 68.1% of patients completed all cycles. The most common reasons for early discontinuation were persistent asymptomatic leukopenia in women aged ≤ 50 years, and limiting infections in women aged > 50 years. Leukopenia was more likely to occur after the third cycle. Significantly worse survival was observed for post-radiochemotherapy elevated C-reactive-protein and lactate dehydrogenase levels, low pre-radiochemotherapy nutritional index, and raised C-reactive-protein-levels; the latter were also predictable for local control. The Glasgow prognostic score did not reliably predict survival. **Conclusion:** Incomplete application of simultaneous chemotherapy leads to inferior local control, and age-dependent limiting factors should be identified at an early stage. In addition to classical risk factors, serological markers (C-reactive-protein, lactate dehydrogenase, nutritional index) show prognostic significance.

## Introduction

Cervical cancer accounts for both, the fourth most frequently diagnosed cancer and the fourth leading cause of cancer death in women with 604 000 new cases and 342 000 deaths in 2020.^1^ The implementation of vaccination against human papilloma virus and screening methods effectively led to the reduction of incidence and mortality, as well as the detection of earlier stages.^2–4^ In managing early-stage cervical cancer, defined as Federation of Gynecology and Obstetrics (FIGO) stages 1A-1B, fertility-sparing surgery or radical hysterectomy represents the cornerstone of curative therapy, whereas for higher stages, definitive radiochemotherapy (RCHT) is the treatment of choice.^5–7^ Overall survival (OS) rates decrease with higher stages, from 4-year OS rates of approximately 98.2% and 97.0% for FIGO stages 1A-1B1, to 83.1% for stage IB3, and poor 5-year OS outcomes of 46.0% for stage IIIA and 5.1% for stage IVA.^8–10^

In addition to FIGO stage, positive nodal status, deep stromal and lymphovascular space invasion (LVSI), histologic subtype of adenocarcinoma, and higher grade are considered high-risk factors, and appear more often in larger tumors.^11–16^ For FIGO 1A, there is a risk for the presence of pelvic lymph node metastases of only 3.5% to 7.4%, and the presence of LVSI significantly reduces tumor control.^8–17^ The need for adjuvant therapy after surgery for early-stage cervical cancer with unfavorable pathological criteria has been widely investigated with proof of superior local control (LC) and progression-free survival, when postoperative RCHT was applied compared to surgery alone.^15,18,19^ This has been proven for high-risk “Peters” criteria with positive lymph nodes, involved surgical margins or parametrial infiltration and intermediate-risk “Sedlis” criteria with large tumors > 4 cm, deep stromal infiltration or lymphangiosis.^18,19^ The addition of postoperative concurrent chemotherapy showed significantly improved progression-free and OS in the adjuvant high-risk setting in the Gynecologic Oncology Group (GOG 109) trial, and concurrent cisplatin-based schemes are currently the standard of care as it has the least toxicity among various chemotherapy regimens.^19–21^ Nevertheless, there is evidence that only patients with multiple lymph nodes or histologic subtype of squamous cell carcinoma may benefit from adjuvant chemotherapy.^12,22^ The assessment of risk factors remains controversial and heterogeneous, and the STARS trial^23^ further questions the sequence of the optimal chemotherapy administration, preferring a sequential application more than concurrent regimens. Furthermore, late toxicity encompassing proctitis, diarrhea, bowel inflammation, fistula or stenosis, ulceration, and toxicity within the urinary bladder, such as cystitis, is significantly higher in patients with trimodality treatment (surgery, radiotherapy [RT], chemotherapy) with severe events in 7% to 15.3% of patients, compared to surgery plus RT alone in 2% to 5.5% of patients;^11,12^ thus, critical patient selection for prevention of overtreatment or undertreatment is still needed.

In terms of optimizing treatment and prognosis prediction, additional prognostic biomarkers with serologic data and the systemic inflammatory immune response focusing on preoperative peripheral blood cell ratios have emerged, showing prognostic values of neutrophil, monocyte, and lymphocyte ratios for predicting OS in cervical cancer.^24,25^ Furthermore, serological laboratory markers with elevated lactate dehydrogenase (LDH) levels have been shown to be associated with inferior survival in cervical cancer and endometrial cancer.^26,27^ In this study, we aimed to evaluate the impact and effectiveness of concurrent cisplatin-based chemotherapy on oncologic outcomes, and investigate the toxicity profile of high-risk subgroups to improve multimodal treatment. Furthermore, we aimed to explore the impact of additional prognostic serologic biomarkers and provide new evidence of serological markers at different time points during RCHT treatment.

## Materials and Methods

In this single-center retrospective study, we analyzed women, who were treated with postoperative radiotherapy and simultaneous weekly cisplatin for cervical cancer between October 2010 and August 2021. The study was conducted according to the guidelines of the Declaration of Helsinki, and approved by the local ethical review board (approval number: S-453/2021). The requirement for informed consent from each individual was waived by the appropriate institutional review board. All patient details and personally identifiable information were de-identified.

Only patients in an adjuvant curative setting with upfront oncologic surgery including hysterectomy and at least pelvic lymphadenectomy, application of postoperative pelvic RT plus simultaneous chemotherapy with weekly cisplatin, and complete laboratory data were included. Treatment concepts were determined in multidisciplinary tumor conferences according to each patient's individual risks and cases with neoadjuvant chemotherapy or palliative treatment regimens were excluded. Patient, treatment and oncologic data were individually assessed. Staging included computed tomography (CT) of the thorax and magnetic resonance imaging of the pelvis, with further classification according to the FIGO 2018 staging system^5^ and eighth edition of the TNM American Joint Committee on Cancer staging system.

### Radiotherapy

External beam radiotherapy (EBRT) was delivered using 6 MV photons and intensity-modulated radiotherapy (IMRT) alone or in combination with brachytherapy (BT). Contouring was performed on 3 mm slice thickness CT planning imaging and delineation according to guidelines and adapted to each patients’ individual risk. Dose constraints for adjacent organs at risk were in accordance with the Quantec recommendations.^28,29^ The clinical tumor volume (CTV) included the pelvic region with the vaginal cuff, the upper vagina, parametrial and paravaginal tissues and nodal irradiation consisting of external and internal iliac, common iliac, obturator, and presacral regions up to the bifurcation of the aorta. The para-aortic region was only included in case of para-aortic lymph metastases. A planning target volume (PTV) with a margin of 0.5 to 2 cm to the CTV was added. The prescribed dose to the PTV for EBRT was 45 to 54 Gy delivered once daily in 25 to 30 fractions, for a treatment time of 5 to 6 weeks. For macroscopic lymph node metastases, a simultaneously integrated boost (SIB) of 54 to 58.8 Gy was applied. Women with high-risk factors, such as lymphangiosis or microscopic residual vaginal tumor, received a vaginal cuff high-dose-rate (HDR) BT, using iridium-192 with an intracavitary single applicator according to Brachytherapy Consensus Guidelines^30^ with single doses of 5 Gy in 1 to 3 fractions. For further comparison, EBRT and HDR brachytherapy boost doses were converted and summed in an equivalent dose in 2 Gy fractions (EQD2) using the linear quadratic model. An α/β ratio of 10 was assumed for the tumor. EQD2 (Gy) = fractional dose × number of fractions × (fractional dose + a/β)/(2 Gy + a/β)].

### Chemotherapy and Peripheral Blood Cell Counts

Cisplatin was administered in a weight-dependent manner (40 mg/m^2^) with 250 ml of intravenous isotonic saline once weekly. The number of intended cycles depended on the duration of EBRT, leading to a total target dose of 200 to 240 mg/m^2^ in 5 to 6 cycles. Supportive antiemetic pre-medication using corticosteroid prophylaxis, granisetron, and aprepitant was given intravenously 2 hours prior to chemotherapy and the following days depending on the individual status and extent of emesis. Before, during, and after chemotherapy, at least 2000 ml of isotonic saline with magnesium, potassium, and 15% mannitol was administered over 6 to 8 hours. Hydration infusion therapy was additionally used in between cycles for nephroprotection.

Patients were assessed for reduced cardiac function, hearing deficits, infections, and general clinical performance status before administration and re-evaluated during the course of treatment. Biochemistry laboratory values and weights were documented before and after each cycle. Dose reduction or hematopoietic stimulation was not routinely performed. Upcoming cycles were omitted or delayed in the presence of persistently low counts of leukocytes (<3/nl), platelets (<100/nl), and symptomatic anemia with hemoglobin levels <8.5 g/dl, in cases of acute kidney failure or inadequate glomerular filtration rate (GFR) using the Cockcroft–Gault formula, fulminant infection, or deterioration of clinical performance status. Routine laboratory data analysis was performed for white cell and platelet counts, hemoglobin levels, and GFR and documented before and after each chemotherapy cycle.

### Serologic Markers and Nutritional Assessment

Baseline and weekly values were assessed before and after RCHT and at each time point of chemotherapy for body weight and body mass index (BMI [kg/m^2^] = weight/height × height), C-reactive protein (CRP), serum albumin, and LDH levels.

We determined the scores of the nutritional index (NI = albumin/CRP)^31^ and the Glasgow prognostic score (GPS)^32^ with grouping of CRP and albumin concentrations into 3 categories with zero points for the best prognosis and values in the normal ranges up to 2 points with the worst prognosis, with elevated CRP combined with hypoalbuminemia.

### Oncologic Outcomes

For each patient, the evaluation of treatment response included follow-up visits with clinical data, referring physician notes, and radiology. Clinical outcomes included the assessment of OS, LC, and distant control (DC). OS was defined as the period from the first day of radiotherapy until the last contact or date of death. LC was considered until any tumor progression at the original site or local pelvic lymph nodes, while DC was defined as metastatic lesions developing outside the pelvis. Common Terminology Criteria for Adverse Events (CTCAE; version 5.0) were used for the grading of acute (<90 days) and late (≥ 90 days) toxicity.

### Statistical Analysis

Kaplan-Meier analysis and the log-rank test or Cox regression were utilized to calculate survival curves and compare subgroups, with statistical significance set at *P* < .05. Univariate and multivariate Cox proportional hazard ratios (HRs) and 95% confidence intervals (CIs) were used to assess the influence of cofactors. Patient and treatment characteristics as well as laboratory data were compared using the Mann–Whitney *U*-test or Pearson chi-square test for continuous or categorical data and analysis of variance with repeated measures and the *t*-test, respectively. Statistical analyses were performed using SPSS (version 28.0; Chicago, Illinois).

## Results

Out of 99 women, who were treated with curative, postoperative RCHT following surgical resection for cervical cancer at our institution between October 2010 and August 2021, 69 patients with a median age of 48 (range: 12-78) years met our inclusion criteria. Pelvic IMRT was applied with a median dose of 45.0 (range: 32.4-54.0) Gy and a median number of 25 (range: 10-30) fractions, with a median SIB dose of 55.5 (range: 54.0-58.8) Gy. One patient had to stop RT after 10 fractions due to infection and refused to continue therapy after recovery. Detailed patient and treatment characteristics are listed in Table 1.

**Table 1.** Patient and Treatment Characteristics.

Characteristics	Women > 50 years (n = 27) Median values (ranges or percentages)	Women ≤ 50 years (n = 42) Median values (ranges or percentages)	Total cohort (n = 69) Median values (ranges or percentages)	*P*-value
**Median age (years)**	60 (51-78)	42.3 (12-50)	48 (12-78)	**<.001**
**FIGO stage**				
1/2	17 (24.7%)	21 (30.4%)	38 (55.1%)	.291
3/4	10 (14.5%)	21 (30.4%)	31 (44.9%)	
**TNM stage**				
1/2	27 (39.1%)	41 (59.5%)	68 (98.6%)	.419
3/4	0 (0%)	1 (1.4%)	1 (1.4%)	
**Nodal status**				
Positive	17 (24.7%)	21 (30.4%)	31 (44.9%)	.291
Negative	10 (14.5%)	21 (30.4%)	38 (55.1%)	
**Resection status**				
Positive	7 (10.2%)	5 (7.2%)	12 (17.4%)	.129
Negative	18 (26.1%)	34 (49.3%)	52 (75.4%)	
**Histological subtype**				
Squamous cell carcinoma	21 (30.4%)	30 (43.5%)	51 (73.9%)	.558
Adenocarcinoma	6 (8.7%)	12 (17.4%)	18 (26.1%)	
**Time from surgery to start of RT (days)**	56 (31-155)	47 (23-95)	49 (23-155)	.085
**Cumulative total dose in EQD2 (α/β = 10) (Gy)**	56.8 (38.1-67.3)	56.8 (44.3-67.3)	56.8 (38.1-67.3)	.483
**Overall RT treatment time (days)**	38 (13-57)	39 (28-52)	38 (13-57)	.936
**Brachytherapy boost**				
Yes	20 (29.0%)	31 (44.9%)	51 (73.9%)	.981
No	7 (10.2%)	11 (15.9%)	18 (26.1%)	
**Simultaneous integrated boost**				
Lymph node metastases	2 (2.9%)	3 (4.3%)	5 (7.2%)	.850
Parametrial tissue	1 (1.4%)	2 (2.9%)	3 (4.3%)	
**Extended radiation field**				
Para-aortic region	0 (0%)	2 (2.9%)	2 (2.9%)	.291
No	27 (39.1%)	40 (58.0%)	67 (97.1%)	
**Karnofsky performance score (%)**	90 (70-100)	90 (70-100)	90 (70-100)	.310
**Baseline body-mass-index**	25.5 (18.2-38.3)	23.0 (15.5-37.1)	24.2 (15.5-38.3)	.263
**Baseline body weight (kg)**	68 (42-108)	65 (33-106)	68 (33-108)	.464
**Baseline glomerular filtration rate (ml/min/1.73 m^2^)**	96 (50-107)	110 (79-178)	105 (50-178)	**<.001**
**Baseline hemoglobin level (g/dL)**	12.4 (8.7-16.3)	12.7 (9.3-14.5)	12.6 (8.7-16.3)	.707
**Baseline leukocyte count (/n**L**)**	7.1 (3.8-14.9)	6.8 (4.4-12.1)	6.9 (3.8-7.4)	.369
**Baseline platelet count (/n**L**)**	313 (215-606)	290 (136-569)	297 (136-606)	.480
**Cisplatin cycles applied as planned**				
Yes	17 (24.6%)	30 (43.5%)	47 (68.1%)	.461
No, omission of cycles	10 (14.5%)	12 (17.4%)	22 (31.9%)	
**Cisplatin cycles**				
< 4 cycles	3 (4.3%)	2 (2.9%)	5 (7.2%)	.318
**≥** 4 cycles	24 (34.8%)	40 (58.0%)	64 (92.8%)	.321
**≥** 5 cycles	21 (30.4%)	38 (55.1%)	59 (85.5%)	.144
**Cisplatin cycles**				
1	1 (1.4%)	1 (1.4%)	2 (2.9%)	
2	1 (1.4%)	0 (0%)	1 (1.4%)	
3	1 (1.4%)	1 (1.4%)	2 (2.9%)	
4	3 (4.3%)	2 (2.9%)	5 (7.2%)	
5	15 (21.7%)	26 (37.7%)	41 (59.4%)	
6	6 (8.7%)	12 (17.4%)	18 (26.1%)	

EQD2: equivalent dose in 2 Gy fractions; FIGO: International Federation of Obstetrics and Gynecology.

Patient and treatment characteristics for patients aged ≤ 50 and > 50 years were comparable in terms of demographic parameters as listed in Table 1, except for significant differences regarding the baseline GFR (*P* < .001), which were significantly lower in older patients.

### Oncologic Outcomes

**Figure 1.**
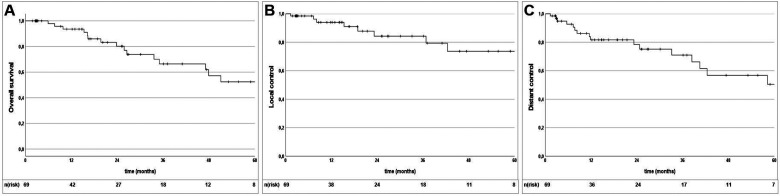
Kaplan-Meier estimates with overall survival (A), local control (B), distant control (C). n(risk): number at risk.

With a median follow-up of 17.7 (range: 1.8-118.7) months, 1-, 2-, 5-year OS rates were 93.6%, 80.2% and 52.5%, respectively (Figure 1A). Univariate analysis revealed a significantly superior OS for younger patients (HR 3.724 [CI: 1.278-10.850], *P* = .016) and women with lower FIGO (HR 1.413 [CI: 0.809-2.468], *P* < .001) and T (HR 3.181 [CI: 1.095-9.237], *P* = .013) stages. Improved survival rates were further revealed in patients without lymphangiosis (HR 0.298 [CI: 0.093-0.955], *P* = .042) and negative resections margins (HR 7.436 [CI: 2.438-22.685], *P* < .001). A lower Karnofsky performance score tended to be slightly related to inferior OS (HR 0.949 [CI: 0.897-1.005], *P* = .076).

Eight local failures (11.6%) were detected with a median time to relapse of 17.1 (range: 1.3-42.4) months, which resulted in 1-, 2-, and 5-year LC rates of 94.0%, 84.3%, and 73.7%, respectively (Figure 1B). In univariate analysis, superior LC was significantly associated with a younger age (≤ 50 years) (HR 4.823 [CI: 0.962-24.190], *P* = .035), with higher numbers of administered chemotherapy cycles (HR 0.577 [CI: 0.0.353-0.942], *P* = .028) and a negative resection margin (HR 8.333 [CI: 1.794-38.715], *P* = .007). Only the number of cisplatin cycles (HR 0.542 [CI: 0.299-0.982], *P* = .043) and resection status (HR 5.870 [CI: 1.127-30.572], *P* = .036) were confirmed as strong independent classical prognostic factors for LC on multivariate analysis.

During the follow-up period, distant metastases were diagnosed in 16 (23.2%) patients, with a median time to failure of 11.6 (range: 1.3-20.0) months with the following organ distribution: pulmonary (n = 8), peritoneal (n = 6), thoracic lymph nodes (n = 5), hepatic (n = 2), skin (n = 2), bone (n = 2), and adrenal (n = 1). The resulting DC rates were 81.7%, 75.2%, and 50.5% at 1, 2, and 5 years, respectively (Figure 1C). Superior DC was shown for younger patients (≤ 50 years) (HR 2.858 [CI: 1.022-7.995], *P* = .045) and a negative resection margin (HR 7.419 [CI: 2.528-21.774], *P* < .001) in univariate analysis, which could be proven in multivariate analysis with inferior DC for patients with positive resection margins (HR 6.195 [CI: 2.005-19.146], *P* = .002).

### Serological Prognostic Factors

Pre-RCHT lowered values of the nutritional index (HR 0.996 [CI: 0.993-0.999], *P* = .016) and raised pre-RCHT CRP levels (HR 1.042 [CI: 1.013-1.072], *P* = .004) were significantly correlated with a worse OS. The GPS tended but did not reliably predict survival (HR: 2.756 [CI: 0.949-8.000], *P* = .062). Factors strongly linked to inferior OS at the end of RT were elevated post-RCHT CRP (HR 1.015 [CI: 1.001-1.030], *P* = .031) and LDH levels (HR 1.010 [CI: 1.000-1.020], *P* = .050). Pre-RCHT raised CRP levels were also predictable for LC (HR 1.040 [CI: 1.001-1.080], *P* = .045). Neither did the pre-post-RCHT ratios of CRP, LDH, and albumin predict OS, LC or DC, nor the ratios of the blood counts.

Table 2 presents a detailed analysis of the prognostic impact on oncologic outcomes.

**Table 2.** Prognostic Value of Serologic Markers at 3 Timepoints: Prior to Surgery, Before and After Radiochemotherapy.

Characteristics	Overall survival		Local control		Distant control	
	HR (95%CI)	*P*	HR (95%CI)	*P*	HR (95%CI)	*P*
**Hemoglobin level**						
Prior to surgery	0.806 (0.488-1.331)	.398	0.857 (0.437-1.682)	.655	0.973 (0.621-1.526)	.906
Pre-RCHT	0.956 (0.639-1.430)	.826	0.859 (0.500-1.478)	.584	1.104 (0.729-1.671)	.640
Post-RCHT	1.361 (0.660-2.806)	.404	1.740 (0.430-7.043)	.438	1.249 (0.635-2.456)	.519
**Leukocyte count**						
Prior to surgery	0.990 (0.956-1.026)	.584	0.987 (0.913-1.066)	.732	1.004 (0.995-1.013)	.417
Pre-RCHT	1.141 (0.904-1.441)	.267	1.074 (0.773-1.490)	.672	1.162 (0.943-1.432)	.160
Post-RCHT	1.174 (0.864-1.597)	.305	1.298 (0.759-2.219)	.342	1.252 (0.856-1.832)	.246
**Platelet count**						
Prior to surgery	1.001 (0.997-1.005)	.551	1.002 (0.995-1.008)	.635	1.000 (0.995-1.004)	.852
Pre-RCHT	1.003 (0.998-1.008)	.292	1.004 (0.997-1.011)	.263	0.999 (0.993-1.005)	.717
Post-RCHT	1.003 (0.992-1.015)	.558	1.011 (0.987-1.036)	.367	0.925 (0.112-7.629)	.943
**LDH**						
Prior to surgery	0.992 (0.973-1.011)	.408	0.993 (0.995-1.031)	.703	0.972 (0.873-1.083)	.610
Pre-RCHT	1.003 (0.990-1.017)	.625	0.993 (0.972-1.016)	.560	0.998 (0.984-1.012)	.799
Post-RCHT	1.010 (1.000-1.020)	**.050**	0.997 (0.977-1.017)	.744	1.005 (0.995-1.015)	.321
**CRP**						
Prior to surgery	1.037 (0.989-1.088)	.134	1.009 (0.962-1.057)	.722	1.001 (0.964-1.039)	.973
Pre-RCHT	1.042 (1.013-1.072)	**.004**	1.040 (1.001-1.080)	**.045**	1.028 (0.999-1.058)	.061
Post-RCHT	1.015 (1.001-1.030)	**.031**	1.006 (0.980-1.032)	.670	1.013 (1.000-1.027)	.052
**Albumin**						
Prior to surgery	0.821 (0.408-1.653)	.581	10.192 (0.001-77230)	.610	0.732 (0.301-1.782)	.492
Pre-RCHT	1.015 (0.765-1.349)	.916	0.922 (0.650-1.308)	.650	0.851 (0.673-1.077)	.179
Post-RCHT	0.972 (0.731-1.293)	.847	0.939 (0.642-1.374)	.747	0.964 (0.771-1.203)	.743
**Glasgow prognostic score**						
Prior to surgery	3.136 (0.322-30.559)	.325	0.948 (0.079-11.361)	.967	1.264 (0.212-7.521)	.797
Pre-RCHT	2.756 (0.949-8.000)	.062	2.906 (0.650-13.004)	.163	1.919 (0.652-5.648)	.237
Post-RCHT	2.477 (0.663-9.250)	.177	6.667 (0.693-64.176)	.101	1.857 (0.584-5.906)	.294
**Nutritional index**						
Prior to surgery	1.023 (0.819-1.297)	.841	1.397 (0.386-5.056)	.610	1.023 (0.819-1.279)	.841
Pre-RCHT	0.996 (0.993-0.999)	**.016**	0.997 (0.993-1.001)	.146	0.998 (0.996-1.001)	.163
Post-RCHT	1.000 (0.997-1.003)	.858	0.998 (0.992-1.003)	.424	1.000 (0.998-1.003)	.886
**Pre–post-ratio hemoglobin**	1.095 (0.025-47.886)	.963	0.330 (0.002-57.489)	.674	4.539 (0.100-206.1)	.437
**Pre–post-ratio leukocyte**	1.225 (0.636-2.359)	.544	1.064 (0.390-2.905)	.903	1.214 (0.628-2.345)	.564
**Pre–post-ratio platelet**	0.753 (0.250-2.266)	.613	0.546 (0.114-2.617)	.449	0.387 (0.119-1.262)	.115
**Pre–post-ratio CRP**	1.027 (0.985-1.071)	.214	0.955 (0.768-1.189)	.682	1.002 (0.965-1.041)	.904
**Pre–post-ratio LDH**	0.871 (0.148-5.113)	.878	0.537 (0.037-7.836)	.649	1.430 (0.282-7.245)	.666
**Pre–post-ratio albumin**	13.568 (0.051-3616)	.360	10.177 (0.02-44010)	.587	5.974 (0.165-216.1)	.329

CI: confidence interval; CRP: C-reactive protein; HR: hazard ratio; LDH: lactate dehydrogenase; RCHT: radiochemotherapy.

### Toxicity

**Figure 2.**
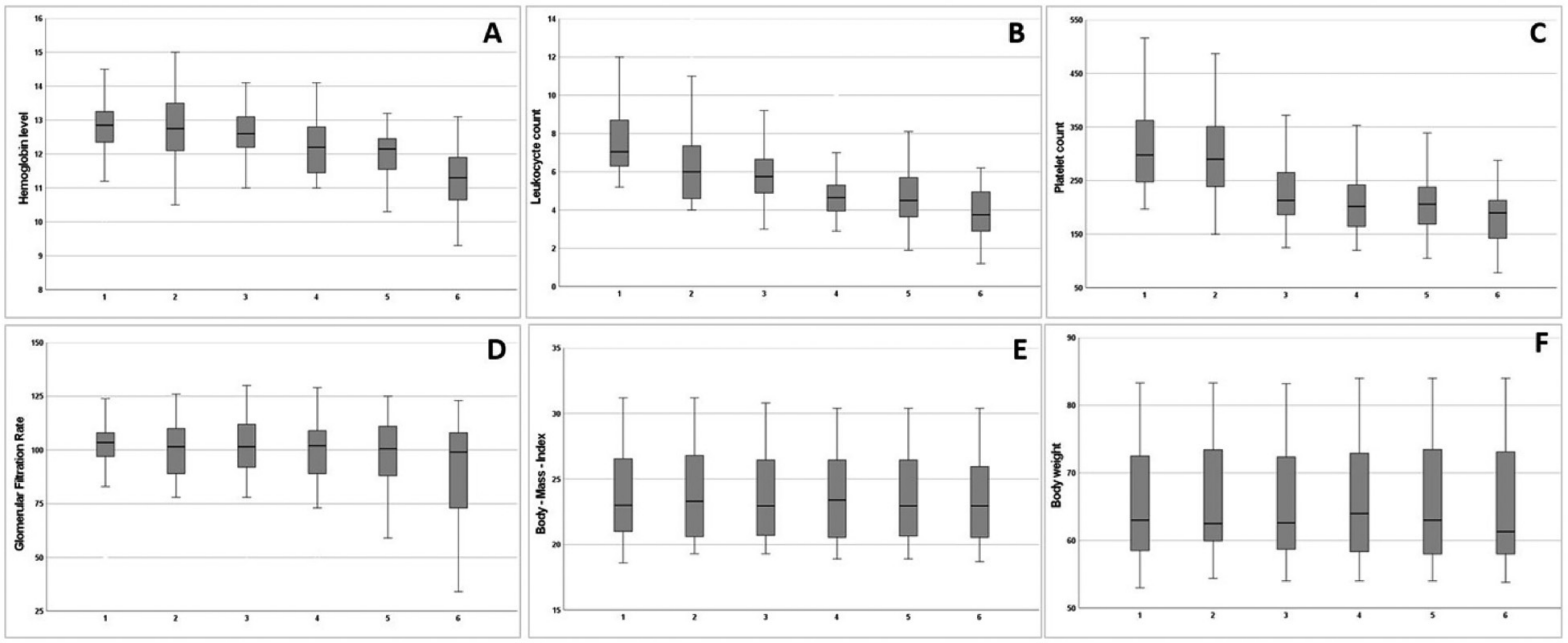
Boxplots with first and third quartiles, minimum, maximum and median values of peripheral blood cell counts with hemoglobin levels (g/dl) (A), leukocyte count (per nl) (B), platelet count (per nl) (C), glomerular filtration rate (ml/min/1.73 m^2^) (D), body mass index (kg/m^2^) (E), and absolute body weight (kilogram) (F) at the timepoint of each cycle.

Figure 2 shows the values of hemoglobin levels, leukocyte and platelet counts, BMI, body weight, and GFR at baseline at the time point of cycle one and over the course of cisplatin RCHT treatment.

Absolute hemoglobin levels (Figure 2A) did not change significantly at a certain time point, but there was a trend for lower levels in the sixth cycle (*P* = .092). Anemia appeared significantly more often at the fourth cycle (*P* < .001). During the course of RCHT, 21 patients had grade 1 (30.4%, 10-10.9 g/dl), 9 patients grade 2 (13.0%, 8-9.9 g/dl), and 1 woman grade 3 (1.4%, 6.5-7.9 g/dl) anemia. In 3 patients red blood cell transfusion was necessary.

Absolute leukocyte counts (Figure 1B) were significantly (*P* < .05) reduced from the start of the first up to the fourth cycle, while leukopenia significantly appeared more often at and after the third cycle (*P* < .001). Twenty women (29.0%) had CTC grade 1 (3-3.9/nl), 22 (31.9%) had grade 2 (2-2.9/nl), and 4 (5.8%) women grade 3 (1-1.9/nl) leukopenia during RCHT. Application of granulocyte colony-stimulating factor was mandatory in one patient with severe symptomatic leukopenia.

Platelet counts (Figure 1C) significantly decreased after the second cycle (*P* = .047), while thrombocytopenia (grade 1, platelets below 100/nl) was only present in 3 patients (4.3%) in the sixth cycle (*P* < .001). None of the patients underwent platelet transfusions. There was no higher grade (≥2 grade as defined by ≤74.9/nl) thrombocytopenia present during chemoradiation.

GFR (Figure 1D) was significantly reduced at the time point of the sixth cycle (*P* = .009). Of note, renal failure limited the application of chemotherapy in 2 patients, while 4 patients suffered from acute renal failure after the end of treatment after completion of all intended cycles (5 cycles: n = 3, 6 cycles: n = 1), leading to chronic renal failure in 2 cases. No significant changes in the BMI (Figure 1E) or absolute body weight (Figure 1F) were observed over the course of RCHT treatment.

The comparison of values at baseline and after the last chemotherapy cycle for the whole cohort showed a median pre-/post-treatment reduction of −9.5% (range: −38.8% to  + 37.9%) for hemoglobin levels, −48.9% (range −81.2% to + 94.8%) for leukocyte counts, −41.1% (range: −66.3% to + 61.0%) for platelet counts, −1.4% (range: −64.9% to  + 16.7%) for the GFR and −0.9% (range: −11.6% to  + 8%) for the BMI and body weight. The reduction in GFR was significantly higher (*P* = .036) in older patients over 50 years than in the younger cohort. The median values and ranges of the absolute and percentage changes in laboratory data for each age-dependent subgroup are listed in detail in Table 3.

**Table 3.** Median Changes of Laboratory Data Over the Course of Cisplatin Treatment from Baseline to End of Treatment Divided Into the 2 Age Groups (>50 Years and ≤ 50 Years).

Characteristics	Women > 50 years Median absolute and percentage changes (range)	Women ≤ 50 years Median absolute and percentage changes (range)	*P*-value
			
**Hemoglobin level**	−1.1 (range: −3.1 to + 3.3) g/dl	−1.3 (range: −5.0 to + 3.5) g/dl	*P* = .694
	−8.7 (range: −23.5 to + 37.9) %	−10.1 (range: −38.8 to + 37.6) %	

**Leukocyte count**	−3.0 (range: −9.2 to + 5.5) /nl	−3.3 (range: −6.5 to + 2.4) /nl	*P* = .653
	−44.0 (range: −81.2 to + 94.8) %	−49.8 (range: −76.9 to + 42.1) %	
**Platelet count**	−116 (range: −400 to + 147) /nl	−128 (range: −278 to + 25) /nl	*P* = .922
	−43.7 (range: −66.0 to + 61.0) %	−40.7 (range: −66.3 to + 15.4) %	
**GFR**	−7 (range: −63 to + 13) ml/min/1.73 m^2^	0.0 (range: −47 to + 16) ml/min/1.73 m^2^	*P* ** = .036**
	−9.6 (range: −64.9 to + 14.3) %	0.0 (range: −38.5 to + 16.7) %	
**BMI**	−0.5. (range: −2.4 to + 1.6) kg/m^2^	0.0 (range: −3.0 to + 2.5) kg/m^2^	*P* = .114
	−1.8 (range: −11.6 to + 5.7) %	0.0 (range: −9.7 to + 8) %	
**Body weight**	−1.5 (range: −6.4 to + 4.3) kg	0.0 (range: −8.5 to + 8.0) kg	*P* = .109
	−1.8 (range: −11.6 to + 5.7) %	0.0 (range: −9.7 to + 8) %	

BMI: body mass index; GFR: glomerular filtration rate.

Administration of 5 cisplatin cycles with a cumulative target dose of 200 mg/m^2^ was intended for 38 patients (55.1%) and 6 cycles with a cumulative target dose of 240 mg/m^2^ for the remaining 31 women (44.9%). Twenty-two patients (31.9%) did not complete this chemotherapy regimen as planned, which was due to one or a combination of the following: persistent leukopenia (n = 12), limiting infections (n = 9), and irreversible renal failure (n = 2). The reasons for early cessation of chemotherapy were significantly age-dependent with asymptomatic persistent leukopenia more often in the younger subgroup (≤50 years, n = 11, *P* = .011) and limiting infections in women over 50 years of age (n = 8, *P* = .011).

Acute low-grade (CTCAE grade 1 + 2) toxicity included urinary disorders (n = 19), diarrhea and proctitis (n = 17), nausea (n = 16), fatigue (n = 15), dermatitis (n = 10), abdominal pain (n = 9), and reversible hearing impairment (n = 5). The latter did not lead to a reduction in chemotherapy, but a delay in chemotherapy application in one patient. Severe acute toxicity (CTCAE grade 3) consisted of gastrointestinal disorders (n = 7, 10.1%) and urinary impairments (n = 3, 4.3%). No higher grade (CTCAE grade 4) toxicity was observed.

## Discussion

Avoiding toxicity in the trimodality concept is an important issue in curative adjuvant RCHT treatment for cervical cancer. The application of simultaneous chemotherapy can lead to severe side effects, while omission can limit oncologic outcome. This study aimed to identify high-risk subgroups and evaluate toxicity profiles and oncologic outcome. Our results underline the importance of age-dependent monitoring of chemotherapy-limiting toxicity profiles of women in the adjuvant treatment of cervical cancer and provide new quantitative evidence on weekly reductions in peripheral blood cell counts and an analysis of prognostic serologic markers and indices as predictive tools that have so far not been assessed for applicability in this entity.

As the impact of heterogonous risk factors on prognosis remains controversial, the inclusion of personalized biomarkers is of great interest to provide more accurate information on prognosis and tracking of therapy. Currently, there are no such strongly validated tumor markers for widespread clinical use for cervical cancer. The evaluation of a causal link between elevated biomarkers due to consecutive alterations in tumor microenvironment and inferior outcome and thus the inclusion of serologic markers is of great interest for prognosis stratification.

Lower pretreatment LDH levels have recently been shown to highly reflect a patient's superior prognosis in the treatment of small cell lung cancer undergoing platinum chemotherapy, in patients with head and neck tumors and lymph node positive prostate cancer as well as uterine cervical cancer.^26,33–35^ In our study a higher LDH level at the end of RCHT treatment was a predictive marker for inferior OS; interestingly, this could not be proven for baseline or pre-RCHT LDH levels. Previous studies have hypothesized that the metabolic cause of elevated LDH levels in patients with poorer outcomes is related to the presence of increased hypoxia due to the enzyme's function in anaerobic conditions.^34,36^

In more detail, elevated serum LDH release in cancer patients has been shown to be correlated with LDH-isoenzyme-5 expression in tumor tissue; furthermore, increased LDH-isoenzyme-5 expression is linked to higher chemo- and radiotherapy resistance and has been reported to be an independent negative prognostic marker in endometrial cancer.^27,37,38^ In addition, the connections between anemia and tumor hypoxia to tumor progression, especially in the context of radiotherapy and tissue microenvironements are highly important for radio- and chemotherapy resistance.^38^ Hemoglobin levels of 12 to 14 g/dl have been considered optimal for tumor therapy, but reliable oxygen measurement tools have so far not been described.^39^ Our study did not find any association between hemoglobin levels and tumor control at any time point or at pre–post-RCHT ratios. Regarding locally advanced cervical cancer, preoperative and pre-/post-radiotherapy LDH and CRP levels have been found to be independent markers for OS and progression-free survival in the definitive and neoadjuvant setting and with a higher predictive value in human papillomavirus-positive cervical cancer.^40–43^ However, so far prior studies have not focused on the evidence for a reliable transferability of this to the adjuvant situation as presented in our cohort. The OS in our study was considerably inferior predicted by pre- and post-RCHT CRP levels, post-RCHT LDH levels, and pre-RCHT values of the nutritional index.

Of note, serologic markers cannot be analyzed without focusing on the toxicity of both chemotherapy and radiotherapy and their impact on laboratory changes. The use of 3-dimensional RT techniques using concurrent weekly cisplatin 40 mg/m^2^ resulted in high rates of grade ≥3 acute hematologic toxicity and 19% acute lower gastrointestinal toxicity, but comparable 3-year OS of 90% and LC of 88%.^21^ The application of advanced and widespread pelvic IMRT techniques as used in our study, however, can lower the rates of hematologic toxicity without diminishing the oncologic outcome. The RTOG 0418 trial of Klopp et al^44^ investigated the impact of the RT dose on the hematopoietic stem cell compartment and found an improved bone marrow sparing in a group of women who received postoperative RCHT and radiotherapy for cervical (n = 40) and endometrial cancer (n = 43) to be significantly associated with lower rates of hematotoxicity. Volume-dose constraints to the bone marrow of V40Gy≤37% with a mean dose below 34.2 Gy were suggested to decrease the reduction in peripheral blood cell counts. Interestingly, these hematotoxicities were not linked to the number of chemotherapy cycles applied in the cervical cancer subgroup.

Our cohort consisted of a large subgroup of women with higher stage FIGO stage 3 + 4 (44.9%) patients after surgery, and we confirmed known classical pathologic high-risk factors of lymphangiosis, positive tumor margins, or larger tumors.^18,19^ The resulting 5-year rates of OS and DC were 52.5% and 50.5%, respectively, and thus comparable to prior studies of approximately 51.6% after chemoradiation in high-risk patients, but were overall poor and further improvement for oncologic outcomes is greatly needed.^8,9,11^ In the context of poor DC, the addition of consolidation chemotherapy to adjuvant concurrent RCHT may be the key to further improvement. In the ACTLACC trial,^45^ patients who received consolidation paclitaxel plus carboplatin after RCHT had a significant benefit in systemic recurrence-free survival. Zhong et al^46^ further confirmed the advantage of multiagent consolidation chemotherapy for high-risk cervical cancer patients for disease-free survival and OS, but with an association of increased high-grade rates of myelosuppression. Sun et al^47^ even had to stop a randomized phase III trial for the evaluation of topotecan plus cisplatin in the postoperative setting due to severe hematologic toxicity. The use of a triweekly cisplatin-alone regimen was proven to have the same oncologic outcome, but was more toxic than weekly cisplatin in a study by Lee et al,^48^ while Zhu et al^49^ showed a slightly better outcome for disease-free survival at the cost of significantly more high-grade leukopenia.

The 5-year pelvic recurrence-free survival rate was 77.3% in an analysis by van den Akker et al^11^ and thus in line with the results of our study with a 5-year LC of 73.7%. The same was found with comparable local recurrence rates of 11.6% in our study compared to 13.9% reported by Rotman et al.^15^ As concurrent chemotherapy could be proven as an independent prognostic factor for superior LC, we strongly advocate for the application of full-course cycles, whenever clinically feasible, as meta-analyses have also revealed the effectiveness of postoperative RCHT for improved OS, LC, and DC compared to adjuvant RT alone especially for lymph node-positive patients.^50,51^

While there is still an international consensus on adjuvant RCHT in high-risk patients, the results of the STARS trial of Huang et al^23^ have reopened the controversial discussion regarding the optimal sequence of adjuvant treatment. In their study, sequential chemoradiation led to higher disease-free-survival and a lower risk of cancer death compared to concurrent RCHT regimes. Furthermore, targeted therapies are increasingly dominating the treatment of cervical cancer and could offer additional attractive future treatment options, while the results of randomized prospective trials comparing postoperative radiotherapy versus RCHT are still awaited.^52–54^

Various issues have so far not been resolved regarding the optimal adjuvant multimodality treatment and remain controversial, but overall, there is a need for a reasonable ratio of treatment-induced toxicity that comes along with the addition of consolidation, multiagent or stronger chemotherapy regimens, and targeted therapies. Nonetheless, reluctant approaches concerning the application of chemotherapy seem to threaten oncologic outcomes. In our study, the definition of patients who were at high risk of chemotherapy-induced toxicity was significantly age-dependent. While a deterioration of clinical performance status or hemato- and nephrotoxicity during initial postoperative RCHT led to approximately 83% of the patients receiving at least 5 and 90% at least 4 cycles in a study by Klopp et al,^44^ this was in line with our results of 85.5% and 92.8%, respectively. However, there is evidence that this could be lower in up to 59.5% of patients receiving full-course chemotherapy, as reported by Mell et al.^55^ As an alkylating agent, cisplatin affects the cell cycle and stem cells,^56^ leading to acute hematologic toxicity during RCHT in cervical cancer with grade 0 + 1 / 2 + 3 anemia (86.5% / 13.5%), leukopenia (56.7% / 43.2%), and thrombocytopenia (97.3% / 2.7%) during RCHT,^55^ which were also found in our cohort, showing grade 0 + 1 / 2 + 3 anemia (85.5% / 14.4%), leukopenia (62.3% / 37.7%), and thrombocytopenia (100% / 0%). We further provided new information on the absolute and relative values of blood count reductions during the course of treatment at different time points of chemotherapy cycles, indicating that the severity of cisplatin-induced anemia is maximal at the fourth cycle and that more emphasis must be placed on the prevention of limiting infections in women over 50 years due to significantly decreasing leukocyte counts from cycle 3 onwards.

The primary limitations of our analysis were caused by the retrospective nature of the study design that may impact the findings. As a consequence, our results and statistical measurements have to be interpreted cautiously, especially in the subgroup analyses, due to the small patient cohort, potential methodological issues, or confounding factors. Moreover, study limitations that arise from the retrospective design include, that the currently investigated preoperative blood cell ratios for gynecologic cancers, such as the neutrophil-to-lymphocyte ratio and systemic inflammatory response index,^25,57,58^ could not be reliably assessed or confirmed due to incomplete data availability. Overall, changes or abnormal values in plasma or serum enzymes or isoenzymes and blood counts can be triggered by multicausal conditions including heart, kidney, or liver disease, and must thus be interpreted cautiously. Further systematic prospective research is needed to further validate and analyze the findings and influencing factors.

Even with the improvement and the use of technical developments, the extent of trimodality toxicity in the treatment of cervical cancer is still high, and the overall optimal adjuvant treatment scheme and chemotherapy sequence remains controversial with poor outcomes. Our data offer a new evaluation of various prognostic indices and additional insight into serologic markers and further provide a better understanding of toxicity profiles and changes during postoperative RCHT, which becomes all the more important in the ongoing discussion about the intensification and modification of systemic therapy regimens.

## Conclusion

We demonstrated that full-course adjuvant concurrent RCHT for cervical cancer can improve LC; thus, age-dependent limiting factors should be identified at an early stage. For this, we advocate special emphasis on the occurrence of anemia at the time point of the fourth cycle and limiting infections as well as renal failure in women aged > 50 years. In addition to classic pathological risk factors, serological markers (CRP, LDH, nutritional index) seem a promising reliable tool for the prediction of the oncologic outcome.

## Abbreviations

BMI: body mass index
BT: brachytherapy
CI: confidence intervals
CRP: C-reactive protein
CT: computed tomography
CTCAE: common terminology criteria for adverse events
CTV: clinical tumor volume
DC: distant control
EBRT: external beam radiotherapy
EQD2: equivalent dose in 2 Gy fractions
FIGO: Federation of Gynecology and Obstetrics
GFR: glomerular filtration rate
GPS: Glasgow prognostic score
HDR: high-dose rate
HR: hazard ratios
IMRT: intensity-modulated radiotherapy
LC: local control
LDH: lactate dehydrogenase
LVSI: lymphovascular space invasion
NI: nutritional index
PTV: planning target volume
RCHT: radiochemotherapy
RT: radiotherapy
SIB: simultaneously integrated boost
OS: overall survival

## References

[1] Sung H Ferlay J Siegel RL , et al. Global cancer statistics 2020: GLOBOCAN estimates of incidence and mortality worldwide for 36 cancers in 185 countries. CA Cancer J Clin 2021;71(3):209‐249. doi: 10.3322/caac.21660. PMID: 33538338. 33538338

[2] Mix JM Van Dyne EA Saraiya M Hallowell BD Thomas CC . Assessing impact of HPV vaccination on cervical cancer incidence among women aged 15–29 years in the United States, 1999–2017: an ecologic study. Cancer Epidemiol Biomarkers Prev. 2021;30(1):30‐37. doi: 10.1158/1055-9965.EPI-20-0846. PMID: 33082207; PMCID: PMC7855406. 33082207 PMC7855406

[3] Vaccarella S Franceschi S Zaridze D , et al. Preventable fractions of cervical cancer via effective screening in six Baltic, central, and eastern European countries 2017-40: a population-based study. Lancet Oncol. 2016;17(10):1445‐1452. doi: 10.1016/S1470-2045(16)30275-3. PMID: 27567054; PMCID: PMC5052457. 27567054 PMC5052457

[4] Jansen EEL Zielonke N Gini A , et al. de Kok IMCM; EU-TOPIA consortium. Effect of organised cervical cancer screening on cervical cancer mortality in Europe: a systematic review. Eur J Cancer. 2020;127:207‐223. doi: 10.1016/j.ejca.2019.12.013. PMID: 31980322. 31980322

[5] Bhatla N Aoki D Sharma DN Sankaranarayanan R . Cancer of the cervix uteri. Int J Gynaecol Obstet. 2018;143(Suppl 2):22‐36. doi: 10.1002/ijgo.12611. PMID: 30306584. 30306584

[6] Landoni F Maneo A Colombo A , et al. Randomised study of radical surgery versus radiotherapy for stage Ib-IIa cervical cancer. Lancet. 1997;350(9077):535‐540. doi: 10.1016/S0140-6736(97)02250-2. PMID: 9284774. 9284774

[7] Rob L Skapa P Robova H . Fertility-sparing surgery in patients with cervical cancer. Lancet Oncol. 2011;12(2):192‐200. doi: 10.1016/S1470-2045(10)70084-X. PMID: 20619737. 20619737

[8] Wenzel HHB Van Kol KGG Nijman HW , et al. Cervical cancer with ≤5 mm depth of invasion and >7 mm horizontal spread - Is lymph node assessment only required in patients with LVSI? Gynecol Oncol. 2020;158(2):282‐286. doi: 10.1016/j.ygyno.2020.04.705. PMID: 32381363. 32381363

[9] Matsuo K Machida H Mandelbaum RS Konishi I Mikami M . Validation of the 2018 FIGO cervical cancer staging system. Gynecol Oncol. 2019;152(1):87‐93. doi: 10.1016/j.ygyno.2018.10.026. PMID: 30389105; PMCID: PMC7528458. 30389105 PMC7528458

[10] Espenel S Garcia MA Langrand-Escure J , et al. Special focus on stage IV cervical cancer patients: a decade experience. Oncology. 2019;97(3):125‐134. doi: 10.1159/000500025. PMID: 31266037. 31266037

[11] van den Akker MJE Horeweg N Beltman JJ Creutzberg CL Nout RA . Efficacy and toxicity of postoperative external beam radiotherapy or chemoradiation for early-stage cervical cancer. Int J Gynecol Cancer. 2020;30(12):1878‐1886. doi: 10.1136/ijgc-2019-001131. PMID: 32591371. 32591371

[12] Dávila Fajardo R van Os R Buist MR , et al. Post-operative radiotherapy in patients with early stage cervical cancer. Gynecol Oncol. 2014;134(1):52‐59. doi: 10.1016/j.ygyno.2014.04.045. PMID: 24784874. 24784874

[13] Park JY Kim DY Kim JH Kim YM Kim YT Nam JH . Outcomes after radical hysterectomy according to tumor size divided by 2-cm interval in patients with early cervical cancer. Ann Oncol. 2011;22(1):59‐67. doi: 10.1093/annonc/mdq321. PMID: 20595451. 20595451

[14] Delgado G Bundy B Zaino R Sevin BU Creasman WT Major F . Prospective surgical-pathological study of disease-free interval in patients with stage IB squamous cell carcinoma of the cervix: a Gynecologic Oncology Group study. Gynecol Oncol. 1990;38(3):352‐357. doi: 10.1016/0090-8258(90)90072-s. PMID: 2227547. 2227547

[15] Rotman M Sedlis A Piedmonte MR , et al. A phase III randomized trial of postoperative pelvic irradiation in Stage IB cervical carcinoma with poor prognostic features: follow-up of a gynecologic oncology group study. Int J Radiat Oncol Biol Phys. 2006;65(1):169‐176. doi: 10.1016/j.ijrobp.2005.10.019. PMID: 16427212. 16427212

[16] Farley JH Hickey KW Carlson JW Rose GS Kost ER Harrison TA . Adenosquamous histology predicts a poor outcome for patients with advanced-stage, but not early-stage, cervical carcinoma. Cancer. 2003;97(9):2196‐2202. doi: 10.1002/cncr.11371. PMID: 12712471. 12712471

[17] Buckley SL Tritz DM Van Le L Jr. , et al. Lymph node metastases and prognosis in patients with stage IA2 cervical cancer. Gynecol Oncol. 1996;63(1):4‐9. doi: 10.1006/gyno.1996.0268. PMID: 8898159. 8898159

[18] Sedlis A Bundy BN Rotman MZ Lentz SS Muderspach LI Zaino RJ . A randomized trial of pelvic radiation therapy versus no further therapy in selected patients with stage IB carcinoma of the cervix after radical hysterectomy and pelvic lymphadenectomy: a Gynecologic Oncology Group Study. Gynecol Oncol. 1999;73(2):177‐183. doi: 10.1006/gyno.1999.5387. PMID: 10329031. 10329031

[19] Peters WA 3rd Liu PY Barrett RJ 2nd , et al. Concurrent chemotherapy and pelvic radiation therapy compared with pelvic radiation therapy alone as adjuvant therapy after radical surgery in high-risk early-stage cancer of the cervix. J Clin Oncol. 2000;18(8):1606‐1613. doi: 10.1200/JCO.2000.18.8.1606. PMID: 10764420. 10764420

[20] Fu ZZ Li K Peng Y , et al. Efficacy and toxicity of different concurrent chemoradiotherapy regimens in the treatment of advanced cervical cancer: a network meta-analysis. Medicine (Baltimore). 2017;96(2):e5853. doi: 10.1097/MD.0000000000005853. PMID: 28079819; PMCID: PMC5266181. 28079819 PMC5266181

[21] Isohashi F Takano T Onuki M , et al. A multi-institutional observational study on the effects of three-dimensional radiotherapy and weekly 40-mg/m2 cisplatin on postoperative uterine cervical cancer patients with high-risk prognostic factors. Int J Clin Oncol. 2019;24(5):575‐582. doi: 10.1007/s10147-018-01380-z. PMID: 30580379; PMCID: PMC6469659. 30580379 PMC6469659

[22] Monk BJ Wang J Im S , et al. Gynecologic Oncology Group; Southwest Oncology Group; Radiation Therapy Oncology Group. Rethinking the use of radiation and chemotherapy after radical hysterectomy: a clinical-pathologic analysis of a Gynecologic Oncology Group/Southwest Oncology Group/Radiation Therapy Oncology Group trial. Gynecol Oncol. 2005;96(3):721‐728. doi: 10.1016/j.ygyno.2004.11.007. PMID: 15721417. 15721417

[23] Huang H Feng YL Wan T , et al. Effectiveness of sequential chemoradiation vs concurrent chemoradiation or radiation alone in adjuvant treatment after hysterectomy for cervical cancer: the STARS phase 3 randomized clinical trial. JAMA Oncol. 2021;7(3):361‐369. doi: 10.1001/jamaoncol.2020.7168. PMID: 33443541; PMCID: PMC7809615. 33443541 PMC7809615

[24] Zheng RR Huang M Jin C , et al. Cervical cancer systemic inflammation score: a novel predictor of prognosis. Oncotarget. 2016;7(12):15230‐15242. doi: 10.18632/oncotarget.7378. PMID: 26885692; PMCID: PMC4924782. 26885692 PMC4924782

[25] Chao B Ju X Zhang L Xu X Zhao Y . A novel prognostic marker systemic inflammation response index (SIRI) for operable cervical cancer patients. Front Oncol. 2020;10:766. doi: 10.3389/fonc.2020.00766. PMID: 32477958; PMCID: PMC7237698. 32477958 PMC7237698

[26] Ye Y Chen M Chen X Xiao J Liao L Lin F . Clinical significance and prognostic value of lactate dehydrogenase expression in cervical cancer. Genet Test Mol Biomarkers. 2022;26(3):107‐117. doi: 10.1089/gtmb.2021.0006. PMID: 35349377; PMCID: PMC8982136. 35349377 PMC8982136

[27] Giatromanolaki A Sivridis E Gatter KC Turley H Harris AL Koukourakis MI ; Tumour and Angiogenesis Research Group. Lactate dehydrogenase 5 (LDH-5) expression in endometrial cancer relates to the activated VEGF/VEGFR2(KDR) pathway and prognosis. Gynecol Oncol. 2006;103(3):912‐918. doi: 10.1016/j.ygyno.2006.05.043. PMID: 16837029. 16837029

[28] Small W Jr Bosch WR Harkenrider MM , et al. NRG Oncology/RTOG consensus guidelines for delineation of clinical target volume for intensity modulated pelvic radiation therapy in postoperative treatment of endometrial and cervical cancer: an update. Int J Radiat Oncol Biol Phys. 2021;109(2):413‐424. doi: 10.1016/j.ijrobp.2020.08.061. PMID: 32905846; PMCID: PMC7856050. 32905846 PMC7856050

[29] Bentzen SM Constine LS Deasy JO , et al. Quantitative analyses of normal tissue effects in the clinic (QUANTEC): an introduction to the scientific issues. Int J Radiat Oncol Biol Phys. 2010;76(3):S3‐S9. doi: 10.1016/j.ijrobp.2009.09.040. PMID: 20171515; PMCID: PMC3431964. 20171515 PMC3431964

[30] Small W Jr Beriwal S Demanes DJ , et al. American Brachytherapy Society. American Brachytherapy society consensus guidelines for adjuvant vaginal cuff brachytherapy after hysterectomy. Brachytherapy. 2012;11(1):58‐67. doi: 10.1016/j.brachy.2011.08.005. PMID: 22265439. 22265439

[31] Alberici Pastore C Paiva Orlandi S González MC . Association between an inflammatory-nutritional index and nutritional status in cancer patients. Nutr Hosp. 2013;28(1):188‐193. doi: 10.3305/nh.2013.28.1.6167. PMID: 23808449. 23808449

[32] Forrest LM McMillan DC McArdle CS Angerson WJ Dunlop DJ . Evaluation of cumulative prognostic scores based on the systemic inflammatory response in patients with inoperable non-small-cell lung cancer. Br J Cancer. 2003;89(6):1028‐1030. doi: 10.1038/sj.bjc.6601242. PMID: 12966420; PMCID: PMC2376960. 12966420 PMC2376960

[33] He M Chi X Shi X , et al. Value of pretreatment serum lactate dehydrogenase as a prognostic and predictive factor for small-cell lung cancer patients treated with first-line platinum-containing chemotherapy. Thorac Cancer. 2021;12(23):3101‐3109. doi: 10.1111/1759-7714.13581. PMID: 34725930. 34725930 PMC8636211

[34] Uehara T Doi H Ishikawa K , et al. Serum lactate dehydrogenase is a predictive biomarker in patients with oropharyngeal cancer undergoing radiotherapy: retrospective study on predictive factors. Head Neck. 2021;43(10):3132‐3141. doi: 10.1002/hed.26814. PMID: 34268826; PMCID: PMC8457164. 34268826 PMC8457164

[35] Blas L Shiota M Yamada S , et al. Lactate dehydrogenase is a Serum prognostic factor in clinically regional lymph node-positive prostate cancer. Anticancer Res. 2021;41(8):3885‐3889. doi: 10.21873/anticanres.15183. PMID: 34281850. 34281850

[36] Höckel M Vaupel P . Tumor hypoxia: definitions and current clinical, biologic, and molecular aspects. J Natl Cancer Inst. 2001;93(4):266‐276. doi: 10.1093/jnci/93.4.266. PMID: 11181773. 11181773

[37] Koukourakis MI Giatromanolaki A Sivridis E , et al. Tumour and Angiogenesis Research Group. Lactate dehydrogenase-5 (LDH-5) overexpression in non-small-cell lung cancer tissues is linked to tumour hypoxia, angiogenic factor production and poor prognosis. Br J Cancer. 2003, 89(5):877‐885. doi: 10.1038/sj.bjc.6601205. PMID: 12942121; PMCID: PMC2394471. 12942121 PMC2394471

[38] Muz B de la Puente P Azab F Azab AK . The role of hypoxia in cancer progression, angiogenesis, metastasis, and resistance to therapy. Hypoxia (Auckl). 2015;3:83‐92. doi: 10.2147/HP.S93413. PMID: 27774485; PMCID: PMC5045092. 27774485 PMC5045092

[39] Vaupel P Mayer A Höckel M . Impact of hemoglobin levels on tumor oxygenation: the higher, the better? Strahlenther Onkol. 2006;182(2):63‐71. doi: 10.1007/s00066-006-1543-7. PMID: 16447012. 16447012

[40] Wang H Wang MS Zhou YH Shi JP Wang WJ . Prognostic values of LDH and CRP in cervical cancer. Onco Targets Ther. 2020;13:1255‐1263. doi: 10.2147/OTT.S235027. PMID: 32103993; PMCID: PMC7023883. 32103993 PMC7023883

[41] Wang WJ Li Y Zhu J Gao MJ Shi JP Huang YQ . Prognostic values of systemic inflammation response (SIR) parameters in resectable cervical cancer. Dose Response. 2019;17(1):1559325819829543. doi: 10.1177/1559325819829543. PMID: 30833874; PMCID: PMC6393952. 30833874 PMC6393952

[42] Li J Wu MF Lu HW Chen Q Lin ZQ Wang LJ . Pretreatment serum lactate dehydrogenase is an independent prognostic factor for patients receiving neoadjuvant chemotherapy for locally advanced cervical cancer. Cancer Med. 2016;5(8):1863‐1872. doi: 10.1002/cam4.779. PMID: 27350066; PMCID: PMC4971915. 27350066 PMC4971915

[43] Jiang Y Gu H Zheng X Pan B Liu P Zheng M . Pretreatment C-reactive protein/albumin ratio is associated with poor survival in patients with 2018 FIGO Stage IB-IIA HPV-positive cervical cancer. Pathol Oncol Res. 2021;27:1609946. doi: 10.3389/pore.2021.1609946. PMID: 34992504; PMCID: PMC8724028. 34992504 PMC8724028

[44] Klopp AH Moughan J Portelance L , et al. Hematologic toxicity in RTOG 0418: a phase 2 study of postoperative IMRT for gynecologic cancer. Int J Radiat Oncol Biol Phys. 2013;86(1):83‐90. doi: 10.1016/j.ijrobp.2013.01.017. PMID: 23582248; PMCID: PMC4572833. 23582248 PMC4572833

[45] Tangjitgamol S Tharavichitkul E Tovanabutra C , et al. A randomized controlled trial comparing concurrent chemoradiation versus concurrent chemoradiation followed by adjuvant chemotherapy in locally advanced cervical cancer patients: ACTLACC trial. J Gynecol Oncol. 2019;30(4):e82. doi: 10.3802/jgo.2019.30.e82. PMID: 31074236; PMCID: PMC6543099. 31074236 PMC6543099

[46] Zhong ML Wang YN Liang MR Liu H Zeng SY . Consolidation chemotherapy in early-stage cervical cancer patients with lymph node metastasis after radical hysterectomy. Int J Gynecol Cancer. 2020;30(5):602‐606. doi: 10.1136/ijgc-2019-000690. PMID: 32156715; PMCID: PMC7362880. 32156715 PMC7362880

[47] Sun W Wang T Shi F , et al. Randomized phase III trial of radiotherapy or chemoradiotherapy with topotecan and cisplatin in intermediate-risk cervical cancer patients after radical hysterectomy. BMC Cancer. 2015;15:353. doi: 10.1186/s12885-015-1355-1. PMID: 25935645; PMCID: PMC4425857. 25935645 PMC4425857

[48] Lee HN Lee KH Lee DW Lee YS Park EK Park JS . Weekly cisplatin therapy compared with triweekly combination chemotherapy as concurrent adjuvant chemoradiation therapy after radical hysterectomy for cervical cancer. Int J Gynecol Cancer. 2011;21(1):128‐136. doi: 10.1097/IGC.0b013e318200f7c5. PMID: 21330837. 21330837

[49] Zhu J Lou R Ji S , et al. Weekly versus triweekly cisplatin-alone adjuvant chemoradiotherapy after radical hysterectomy for stages IB-IIA cervical cancer with risk of recurrence. Anticancer Drugs. 2021;32(2):203‐209. doi: 10.1097/CAD.0000000000001018. PMID: 33186140. 33186140

[50] Yang J Yin J Yan G Huang D Wang J . Postoperative chemoradiotherapy versus radiotherapy alone for cervical cancer: a systematic review and meta-analysis. J Obstet Gynaecol. 2016;36(5):641‐648. doi: 10.3109/01443615.2015.1134458. PMID: 26821995. 26821995

[51] Trifiletti DM Swisher-McClure S Showalter TN Hegarty SE Grover S . Postoperative chemoradiation therapy in high-risk cervical cancer: re-evaluating the findings of gynecologic oncology group study 109 in a large, population-based cohort. Int J Radiat Oncol Biol Phys. 2015;93(5):1032‐1044. doi: 10.1016/j.ijrobp.2015.09.001. PMID: 26581141. 26581141

[52] Colombo N Dubot C Lorusso D , et al. KEYNOTE-826 Investigators. Pembrolizumab for persistent, recurrent, or metastatic cervical cancer. N Engl J Med. 2021;385(20):1856‐1867. doi: 10.1056/NEJMoa2112435. PMID: 34534429. 34534429

[53] GOG 0263. Ryu SY Koh W . Radiation therapy with or without chemotherapy in patients with stage I or stage II cervical cancer who previously underwent surgery. https://clinicaltrials.gov/ct2/show/NCT01101451

[54] Mahmoud O Hathout L Shaaban SG Elshaikh MA Beriwal S Small W Jr. Can chemotherapy boost the survival benefit of adjuvant radiotherapy in early stage cervical cancer with intermediate risk factors? A population based study. Gynecol Oncol. 2016;143(3):539‐544. doi: 10.1016/j.ygyno.2016.10.022. PMID: 27769525. 27769525

[55] Mell LK Kochanski JD Roeske JC , et al. Dosimetric predictors of acute hematologic toxicity in cervical cancer patients treated with concurrent cisplatin and intensity-modulated pelvic radiotherapy. Int J Radiat Oncol Biol Phys. 2006;66(5):1356‐1365. doi: 10.1016/j.ijrobp.2006.03.018. PMID: 16757127. 16757127

[56] Pasetto LM D’Andrea MR Brandes AA Rossi E Monfardini S . The development of platinum compounds and their possible combination. Crit Rev Oncol Hematol. 2006;60(1):59‐75. doi: 10.1016/j.critrevonc.2006.02.003. PMID: 16806960. 16806960

[57] Li YX Chang JY He MY , et al. Neutrophil-to-lymphocyte ratio (NLR) and monocyte-to-lymphocyte ratio (MLR) predict clinical outcome in patients with stage IIB cervical cancer. J Oncol. 2021;2021:2939162. doi: 10.1155/2021/2939162. PMID: 34539781; PMCID: PMC8443385. 34539781 PMC8443385

[58] Huang H Liu Q Zhu L , et al. Prognostic value of preoperative systemic immune-inflammation index in patients with cervical cancer. Sci Rep. 2019;9(1):3284. doi: 10.1038/s41598-019-39150-0. PMID: 30824727; PMCID: PMC6397230. 30824727 PMC6397230

